# Carrier Dynamics Determines the Optimization Strategies of Perovskite LEDs and PVs

**DOI:** 10.34133/research.0112

**Published:** 2023-04-11

**Authors:** Saixue Wang, Yu Cao, Qiming Peng, Wei Huang, Jianpu Wang

**Affiliations:** ^1^ Key Laboratory of Flexible Electronics (KLOFE), Institute of Advanced Materials (IAM) & School of Flexible Electronics (Future Technologies), Nanjing Tech University (NanjingTech), 30 South Puzhu Road, Nanjing 211816, China.; ^2^ Strait Laboratory of Flexible Electronics (SLoFE), Strait Institute of Flexible Electronics (SIFE, Future Technologies), Fujian Normal University, Fuzhou 350117, China.

## Abstract

Metal halide perovskites have advanced greatly in both light-emitting diodes (LEDs) and photovoltaics (PVs) through delicate device engineering. The optimization strategies of perovskite LEDs and PVs have been demonstrated to be quite different. Here, we show that this dissimilarity in device fabrications can be well understood based on the analysis of carrier dynamics in LEDs and PVs.

Metal halide perovskites, which have revolutionized the optoelectronic community during the past decade, have turned out to be a very special semiconductor. Unlike most conventional semiconductors that can only be used in photovoltaic (PV) devices (such as silicon) or light-emitting diode (LED) devices (such as GaN), the perovskites have made great progresses in both applications in PVs and LEDs. The power conversion efficiency of perovskite PVs have raised from the initial value of 3.8% in 2009 to 25.6% certified in 2022 [1,2]. The external quantum efficiencies (EQEs) of perovskite LEDs have exceeded 20%, approaching the commercial organic LED’s levels [3,4]. However, although based on the same material system, the optimization strategies of these 2 kinds of devices have been demonstrated to be quite different.

For perovskite LEDs, defect passivation is of most importance, although this may lead to a declined carrier mobility. The perovskite emitters can be classified to 3-dimensional (3D) perovskites, mixed-dimensional perovskites, and perovskite nanocrystals. In 3D perovskite LEDs, many additives were commonly used to passivate the defects, leading to a high photoluminescence quantum efficiency (PLQE) of above 70% [5]. However, the insulating additives resulted in perovskite films composed of small (even discrete) crystals with largely decreased carrier mobility (Fig. 1A) [3]. In mixed-dimensional perovskite LEDs, massive large size organic cations are used to passivate defects as well as control the distribution of 2D/quasi-2D/3D phases to improve the device performance [4,6], while leading to the inferior carrier transport. In LEDs based on perovskite nanocrystals, additional agents or ligands are commonly required during synthesis [7,8], which will inevitably lead to the deteriorated carrier mobility in the film.

In contrast, to fabricate a high-performance perovskite PV, more attention has been paid to improve the crystallinity of perovskites. The optimization strategies include crystallization control by additives [9], sequential deposition method [10], and vacuum flash-assisted solution processing method [11]. It should be mentioned that all these methods are applied to form dense perovskite films composed of large crystals that are beneficial to carrier transport (Fig. 1B). Although defect passivation has been mentioned frequently, the PLQEs of perovskite films used in PVs were generally much lower than that used in LEDs [12].

Here, we show that the different optimization strategies of perovskite LEDs and PVs can be understood based on the analysis of carrier dynamics (Fig. 1C to F). For a perovskite LED without considering exciton recombination due to the low electron-hole binding energy, if we assume that the carrier injection is balanced and there is no interface issue, the carrier dynamics can be expressed as Eq. 1

$$\frac{\mathrm{dn}}{\mathrm{dt}}=G-k_{T}n-k_{R}n^{2}-k_{A}n^{3}$$ (1)

where *n* is the carrier density, *G* is the carrier injection rate, and *k*_T_, *k*_R_, and *k*_A_ are the rate constants of trap-assisted recombination, bimolecular recombination, and Auger recombination, respectively. The EQE of a LED is proportional to internal quantum efficiency (IQE), i.e., the radiative recombination efficiency of the carriers, *η*_R_, which can be expressed as Eq. 2

$$\mathrm{IQE}=\frac{k_{R}}{\frac{k_{T}}{n}+k_{R}+k_{A}n}$$ (2)

As can be seen, the IQE of a LED is determined by the competition between the radiative recombination and the nonradiative recombination (Fig. 1C).

Figure 1E shows the dependence of the IQE of a perovskite LED on the carrier density for different trap-assisted recombination rates. The IQE increases largely with the decrease in the trap-assisted recombination rate. Thus, to improve the EQE of a LED, we should suppress the nonradiative recombination, especially the trap-assisted recombination, since Auger recombination should not be dominant due to the moderate carrier concentration. This means that defect passivation is of great importance. That is why massive insolating additives had been added to the perovskite film in previous reports, to passivate surface defects or reduce bulk defects by controlling the crystallization process [13], although they will largely reduce the carrier mobility. We note that although lower carrier mobility can limit the brightness of LEDs, usually this issue is not important since the thickness of the perovskite layer in a LED is less than 50 nm, which is an order of magnitude thinner than that in a PV.

For a PV device, the carrier dynamics (Fig. 1D) can be simply expressed as Eq. 3

$$\frac{\mathrm{dn}}{\mathrm{dt}}=G-k_{T}n-k_{R}n^{2}-k_{A}n^{3}-\frac{\mu E}{L}n$$ (3)

Here, *G* is the photocarrier generation rate, the last term in the equation represents the carrier extraction rate, where *μ* is the carrier mobility, *E* is the built-in electric field in the active perovskite layer, and *L* is the thickness of the perovskite layer. Because here we aim to discuss the relationship between the properties of perovskite and the device efficiency, other layers in the PV device are considered to be perfect, that is, they do not absorb light and have no resistance. By neglecting the Auger recombination due to the low carrier density, we can obtain the short-circuit current (*J*_sc_) of a PV as Eq. 4

$$J_{\mathrm{sc}}=\frac{q\frac{\mu E}{L}}{k_{T}+k_{R}n+\frac{\mu E}{L}}\int \frac{P\left(\lambda \right)\lambda }{\mathrm{hc}}\left(1-e^{-\alpha \left(\lambda \right)L}\right)\mathrm{d\lambda}$$ (4)

Here, *q* is the charge of the carriers, *P*(λ) is the intensity of the solar light at AM 1.5G level where λ is the wavelength, *h* is the Plank constant, and *α*(λ) is the absorption coefficient of the perovskite. Obviously, although decreasing the recombination (both nonradiative and radiative) rate is an approach to increase *J*_sc_, a more convenient and effective way is to increase the carrier extraction rate, i.e., improving the carrier transport.

As can be seen in Fig. 1F, *J*_sc_ of a perovskite PV almost remains unchanged when decreasing the trap-assisted recombination rate, while it increases largely with the raise of carrier mobility. This reveals that to optimize a perovskite PV, improving the carrier transport is of primary importance. Generally, the nature of crystal grain boundaries that determines the mobility plays a dominant role in carrier transport at room temperature, which means that dense films composed of big crystals with decreased boundaries are required. This is consistent with the reported high-performance perovskite PVs [9–11].

One might claim that higher trap density in PVs will reduce the carrier mobility and, as a consequence, decreases *J*_sc_ and fill factor (FF). Normally, the carrier mobility is determined by scatterings with defects and phonons. The defect scattering is dominant at low temperature, while the phonon scattering plays the major role at high temperature. Therefore, a moderate increase in trap density would not largely decrease the carrier mobility. This is consistent with literature reports [14,15], where for MAPbBr_3_ (MA is methylamine), the carrier mobility only decreased from 35 to 13 cm^2^ V^–1^ s^–1^ when the trap densities increased from 10^9^ to 10^13^ cm^–3^.

Alternatively, one may argue that a higher trap density will lead to an increase in nonradiative recombination, resulting in a decreased open-circuit voltage (*V*_OC_), and thus decreases the energy conversion efficiency of the device. We have evaluated the impact of the trap-assisted recombination rate on the *V*_OC_ loss for PVs based on FAPbI_3_ (FA is formamidine) by using Eq. 5

$$\Delta V_{\mathrm{OC}}=-\frac{K_{B}T}{q}\text{ln}\left({\mathrm{EQE}}_{\mathrm{LED}}\right)$$ (5)

Here, Δ*V*_OC_ is the nonradiative *V*_OC_ loss, *K*_B_ is the Boltzmann constant, *T* is the temperature, *q* is the charge of the carriers, and EQE_LED_ is the EQE of the PV when working as a LED under current injection that is equivalent to 1 sun illumination, and assumes a 20% light outcoupling efficiency. We can find that when the trap-assisted recombination increases 2 orders of magnitudes (from 10^4^ to 10^6^ s^–1^), the nonradiative *V*_OC_ loss only increases 0.038 V (from 0.042 to 0.08 V), although EQE_LED_ largely decreases from 19.7% to 4.48%. Therefore, a moderate increase in trap density will only slightly decrease the open-circuit voltage of the device.

We have revealed the reason why we choose different optimization paths to achieve high-performance perovskite LEDs and PVs at this stage. However, we understand that carrier transport and defect passivation are both important for the 2 kinds of devices when they move toward the theoretical limited high efficiencies. At that time, the statement that “an excellent perovskite PV is also a good LED” will become a reality [16]. Nonetheless, a perfect LED is not necessarily a good PV, since the LED usually does not require a thick active layer to absorb light.

**Figure.**
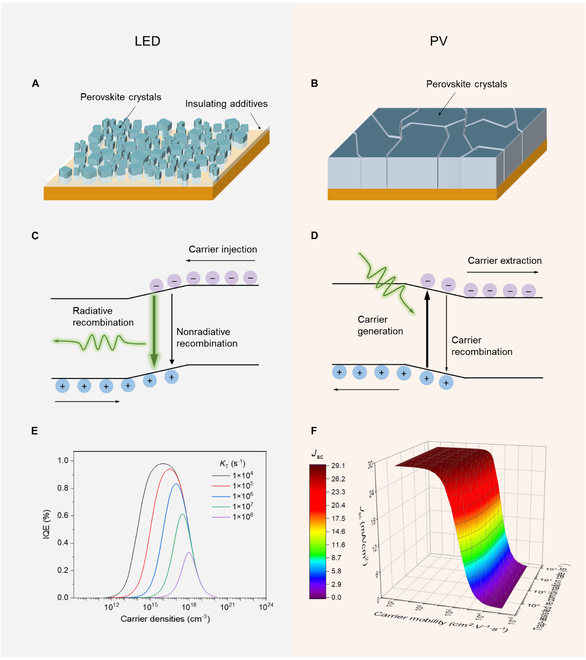
Morphologies of typical perovskite films and charge carrier dynamics in LEDs and PVs. (A) The perovskite film in a LED is commonly made from small (even discrete) grains, which can largely deteriorate carrier transport. (B) The perovskite film in a PV is usually composed of tightly packed large crystals, which is beneficial to carrier transport. (C) Schematic diagram of carrier injection and recombination in a LED. (D) Schematic diagram of carrier generation, recombination, and extraction in a PV. (E) The IQEs of a LED as a function of injected carrier density for different trap-assisted recombination rates. Here, the bimolecular recombination and Auger recombination rates are assumed to be 1 × 10^–10^ cm^3^ s^–1^ and 1 × 10^–28^ cm^6^ s^–1^, respectively (data from [17]). (F) *J*_sc_ of a PV as a function of carrier mobility and trap-assisted recombination rate. In the calculation, the bimolecular recombination rate is 1 × 10^–10^ cm^3^ s^–1^, the Auger recombination is neglected, the perovskite thickness is set to 500 nm, the built-in voltage is assumed to be 1 V that is similar to the open-circuit voltage of perovskite PVs, and the device is under 1 sun illumination.
